# Relaxin-3 Receptor (RXFP3) Signalling Mediates Stress-Related Alcohol Preference in Mice

**DOI:** 10.1371/journal.pone.0122504

**Published:** 2015-04-07

**Authors:** Andrew W. Walker, Craig M. Smith, Berenice E. Chua, Elena V. Krstew, Cary Zhang, Andrew L. Gundlach, Andrew J. Lawrence

**Affiliations:** 1 Neuropeptides Division, The Florey Institute of Neuroscience and Mental Health, Melbourne, Victoria, Australia; 2 Behavioural Neuroscience Division, The Florey Institute of Neuroscience and Mental Health, Melbourne, Victoria, Australia; 3 Florey Department of Neuroscience and Mental Health, The University of Melbourne, Melbourne, Victoria, Australia; 4 Department of Anatomy and Neuroscience, The University of Melbourne, Melbourne, Victoria, Australia; Nathan Kline Institute for Psychiatric Research and New York School of Medicine, UNITED STATES

## Abstract

Stressful life events are causally linked with alcohol use disorders (AUDs), providing support for a hypothesis that alcohol consumption is aimed at stress reduction. We have previously shown that expression of relaxin-3 mRNA in rat brain correlates with alcohol intake and that central antagonism of relaxin-3 receptors (RXFP3) prevents stress-induced reinstatement of alcohol-seeking. Therefore the objectives of these studies were to investigate the impact of Rxfp3 gene deletion in C57BL/6J mice on baseline and stress-related alcohol consumption. Male wild-type (WT) and Rxfp3 knockout (KO) (C57/B6J^RXFP3TM1/DGen^) littermate mice were tested for baseline saccharin and alcohol consumption and preference over water in a continuous access two-bottle free-choice paradigm. Another cohort of mice was subjected to repeated restraint followed by swim stress to examine stress-related alcohol preference. Hepatic alcohol and aldehyde dehydrogenase activity was assessed in mice following chronic alcohol intake and in naive controls. WT and Rxfp3 KO mice had similar baseline saccharin and alcohol preference, and hepatic alcohol processing. However, Rxfp3 KO mice displayed a stress-induced reduction in alcohol preference that was not observed in WT littermates. Notably, this phenotype, once established, persisted for at least six weeks after cessation of stress exposure. These findings suggest that in mice, relaxin-3/RXFP3 signalling is involved in maintaining high alcohol preference during and after stress, but does not appear to strongly regulate the primary reinforcing effects of alcohol.

## Introduction

Alcohol use disorders (AUDs), like other substance use disorders, are characterised in part by recurrent, escalating alcohol use [1], and environmental triggers, including stressful life experiences, can elicit a state of persistent anxiety that promotes the development and maintenance of compulsive alcohol-seeking and consumption. In humans, traumatic events, including life-threatening accidents or severe physical assault, are associated with at-risk drinking and AUDs can emerge following traumatic stress exposure [2]. Rodent models using maternal separation or isolation rearing from weaning also demonstrate that stress exposure has long-lasting effects that can increase first-experience alcohol intake in adult rats [3, 4] and mice [5], consistent with stress-related initiation of alcohol consumption.

Stress is also implicated in the maintenance of alcohol consumption in humans, with progression to compulsive alcohol use thought to be due in part to a shift from positively to negatively reinforced drug-seeking in which alcohol is sought to reduce tension or relieve aversive states such as withdrawal [6]. This hypothesis is supported by research using an acute psychosocial stressor, the Trier Social Stress Test (TSST), which potentiated alcohol drinking in non-treatment seeking alcoholics [7]; and in rodent models of alcohol dependence [8, 9].

Conditional genetic deletion of corticotropin-releasing factor (CRF) receptor-1 (CRF_1_) in extra-hypothalamic regions of adult mouse brain attenuates stress-induced alcohol intake. In contrast, global CRF_1_ gene deletion (knockout) enhances stress-induced alcohol intake [10], highlighting the complex, site-specific mechanisms by which neuropeptides regulate stress and alcohol consumption. The orexin system is also involved in mediating primary and conditioned reinforcing effects of alcohol [11–14], and stress-induced reinstatement of alcohol-seeking [15]. Notably, recent studies in rat have identified the relaxin-3/RXFP3 system as highly stress responsive [16, 17] and involved in the regulation of alcohol self-administration and stress-induced reinstatement of alcohol-seeking [18].

Relaxin-3 is the most recently identified relaxin family peptide [19, 20] and is highly conserved [21] across species including zebrafish [22], mouse [23], rat [24, 25], macaque [26] and human [19]. The majority of relaxin-3 expressing neurons are located in the midline pontine *nucleus incertus* (NI) [20, 27] and innervate widespread forebrain areas [23–25, 28] that express the cognate relaxin-3 receptor, ‘relaxin family peptide 3 receptor’ (RXFP3) [29–31]. CRF_1_ is expressed by relaxin-3 neurons within rodent NI [17, 25], and these relaxin-3 neurons are activated following exposure to various neurogenic stressors in a CRF-dependent manner [16, 25]. Relaxin-3 positive fibres and/or RXFP3 mRNA/binding sites are also present within the amygdala, bed nucleus of the stria terminalis (BNST), periaqueductal grey (PAG), paraventricular thalamic nucleus (PVT), and paraventricular hypothalamic nucleus (PVN) [23–25]. Intracerebroventricular (icv) infusions of relaxin-3 and selective RXFP3 agonists in rat activate PVN CRF neurons and the HPA axis [32], and modulate anxiety and depressive-like behaviour, respectively [33]. Importantly, both central (icv) and intra-BNST infusion of a selective RXFP3 antagonist attenuates alcohol self-administration and stress-induced reinstatement of alcohol-seeking in rat [18], and relaxin-3 mRNA levels in the NI correlate with alcohol intake [34].

Therefore, we aimed to further characterise the role of RXFP3 signalling in alcohol consumption using a germline Rxfp3 knockout (KO) mouse (C57/B6J^RXFP3TM1/DGen^), which display normal health and well-being, with no overt deficits in motor coordination or memory [35]. We report that Rxfp3 KO mice markedly reduce preference for a 10% v/v ethanol solution following multiple stress exposures compared to wild-type (WT) littermates, an effect that once established was persistent.

## Methods

All animal experiments were performed in accordance with the Prevention of Cruelty to Animals Act, 1986 under the guidelines of the National Health and Medical Research Council Code of Practice for the Care and Use of Animals for Experimental Purposes in Australia and approved by the Animal Ethics Committee of The Florey Institute of Neuroscience and Mental Health.

### Animals and Housing

Rxfp3 KO mice (C57/B6J^RXFP3TM1/DGen^) were generated by Deltagen Inc. (San Carlos, CA, USA) and originally supplied by Janssen Pharmaceutical Companies of Johnson & Johnson PR&D LLC (San Diego, CA, USA). F1 129S5:B6 founders were backcrossed onto a C57BL/6J background. Charles River’s Speed Congenics program (Marker-Assisted Accelerated Backcrossing) was used to screen the background genetics of progeny and select those with the highest percentage of C57BL/6J background to mate to produce successive generations until a congenic (~100%) colony was achieved. For further details see [35].

Cohorts of age-matched Rxfp3 KO and WT littermate mice were produced from heterozygous pairings and genotypes were identified using a Sigma REDExtract-N-Amp Tissue PCR Kit (Sigma-Aldrich, Castle Hill, NSW, Australia). Three primers were used for the PCR reaction: WT forward, 5'-GCTCATAGCAGTGAGGAAGAAGACG-3'; WT reverse, 5'-GCTGGTTCTCTACCTGATGAAGAGC-3' and; KO reverse, 5'-GGGCCAGCT CATTCCTCCCACTCAT-3'. After an initialisation step (94°C for 2 min), tail samples were exposed to 30 cycles of PCR. Each cycle included a denaturation (94°C for 30 s), annealing (temperatures of 63°C, 62°C and 60°C for 30 s (2 cycles each for the first 6 cycles) and 58°C for 30 s for the remaining 24 cycles) and elongation (72°C for 1 min) phase. Samples were finally exposed to an additional 10 min of 72°C. This PCR reaction yields 232 bp and/or 461 bp products in the presence of the WT and/or KO alleles, respectively, which were subsequently separated via gel electrophoresis and visualised under ultra violet (UV) light using Invitrogen SYBR safe DNA stain (Life Technologies, Mulgrave, VIC, Australia).

All mice were weaned after 3 weeks and group-housed (maximum 6 per cage, mixed genotypes) in a temperature (~20°C) and humidity (~50%) controlled room on a 12 h light/dark cycle (lights on 0700 h). For two-bottle choice experiments, mice were individually housed at 8–10 weeks of age until study completion. All mice had food and water available *ad libitum*.

Cohort 1 (n = 14 WT/11 KO male mice) and Cohort 2 (n = 17 WT/16 KO male mice) were given *ad libitum* two-bottle choice between 0.1% w/v saccharin and tap water, and alcohol (see below for concentrations) and tap water, respectively. Livers were collected from Cohort 2 and naive control mice to assess endogenous alcohol and aldehyde dehydrogenase activity. Cohort 3 (n = 9 WT/20 KO male mice) was used to investigate the impact of stress on voluntary alcohol consumption in the two-bottle choice paradigm.

### Treatments

Saccharin solutions for two-bottle choice experiments (0.1% w/v, sodium salt hydrate, Sigma-Aldrich) were prepared fresh weekly by dissolving saccharin in tap water. Ethanol solutions for two-bottle choice experiments were prepared fresh weekly by diluting anhydrous absolute ethanol in tap water.

### Two-Bottle Free-Choice Drinking

Two separate cohorts of individually housed mice were acclimatised to drinking from two freely available bottles of tap water for 1 week, after which time one bottle was replaced with either a 0.1% w/v saccharin solution or a 5% v/v ethanol solution for 4 weeks, depending on treatment assignment. Saccharin intake (mL/kg bodyweight), saccharin preference (0.1% saccharin intake [mL]/total fluid intake [mL] × 100) and total fluid intake (mL/kg bodyweight) were measured daily and mice were weighed weekly. Similar formulas were used to calculate preference and total fluid intake of mice consuming alcohol; however alcohol intake was expressed in g/kg bodyweight (alcohol [mL] × alcohol concentration × 0.79/kg bodyweight). Mice were exposed to progressively increasing concentrations of ethanol (5%, 10%, 15% and 20% v/v) with at least 2 weeks at each concentration. All fluids were presented in 250 mL bottles (Tecniplast, Italy) with ball-bearing free sippers, and bottle position was randomly alternated to control for side preference. Bottles were weighed daily between 1000 h and 1200 h to the nearest 0.1 g.

### Liver Preparation

A subset of mice from Cohort 2 (n = 6 WT/6 KO males) matched for alcohol preference and intake (g/kg), and naive controls (n = 5 WT/7 KO males), were used and livers were processed ~1 h after alcohol was removed from the home cages of mice in Cohort 2, as described earlier [36]. Mice were anaesthetised by isoflurane inhalation (IsoFLO; Abbott Laboratories, Melbourne, VIC, Australia) and livers were dissected, weighed wet and frozen over liquid nitrogen for storage (-80°C). Livers were subsequently divided into 4 pieces to increase surface area, incubated for 1 h at 37°C in carbogenated physiological saline solution [(PSS); composition in mM: NaCl, 118.0; KCl, 4.7; NaH_2_PO_4_, 1.0; MgCl_2_, 1.2; CaCl_2_, 1.3; NaHCO_3_, 25.0 and; ethylenediaminetetraacetate (EDTA), 0.04] and manually homogenised in 9 mL ice-cold sucrose buffer (0.25 M sucrose, 5 mM Tris, 0.5 mM EDTA, and 0.5 mM dithiothreitol; pH 7.5). Homogenates were centrifuged (4°C) at 700 *g* for 10 min to remove cellular debris and the resultant supernatant was centrifuged (4°C) at 10,000 *g* for 30 min. The pellet (mitochondrial fraction) was resuspended in 10 mL pyrophosphate buffer (50 mM sodium pyrophosphate; pH 8.8) and divided into 1.5 mL aliquots for storage (-80°C). The supernatant was centrifuged (4°C) at 48,000 *g* for 1 h and the resultant supernatant (cytosolic fraction) was also divided into 1.5 mL aliquots for storage (-80°C). Protein concentration of each sample was determined in duplicate using the recommended manufacturers’ protocol of a Bio-Rad Protein Assay kit (Bio-Rad Laboratories, Gladesville, NSW, Australia).

### Alcohol Dehydrogenase (ADH) Assay

Endogenous hepatic ADH activity was determined by assessing nicotinamide adenine dinucleotide (NADH)-induced increase in absorbance at 340 nm on a microplate spectrophotometer (Bio-Rad Benchmark Plus; Bio-Rad Laboratories) as described [36]. Briefly, the assay was performed in duplicate at 37°C in a final volume of 1.25 mL glycine buffer (0.1 M glycine; pH 10) containing 2.4 mM of β-NAD+ and a volume of cytosolic fraction equivalent to 1 mg protein. Following 5 min of equilibration, the addition of ethanol (0.1–100 mM) initiated the reaction, which was allowed to incubate for 5 min before increased absorbance was measured relative to blank reactions. A concentration-response curve was constructed to determine the concentration of ethanol (10 mM) that produced maximal ADH activity and the ADH inhibitor pyrazole (10 mM) was subsequently used in triplicate to inhibit the reaction induced by 10 mM ethanol, thus testing the specificity of the assay.

### Aldehyde Dehydrogenase (ALDH) Assay

Endogenous hepatic ALDH activity was determined by observing the NADH-induced increase in absorbance at 340 nm on a microplate spectrophotometer (Bio-Rad Benchmark Plus) as described [36]. Briefly, the assay was performed in duplicate at room temperature in a final volume of 1.25 mL pyrophosphate buffer (50 mM sodium pyrophosphate; pH 8.8) containing 1.5 mM β-NAD+; pyrazole (0.1 mM) to block ADH activity; rotenone (2 μM in dimethylsulphoxide: 0.2% final volume) to inhibit mitochondrial NADH oxidase; sodium deoxycholate (0.01% w/v) to increase solution clarity for analysis and release latent activity and; mitochondrial fraction corresponding to 1 mg protein. After 5 min of equilibration, the addition of acetaldehyde (1–1000 mM) initiated the reaction which was then incubated in a sealed 48 well plate for 15 min at room temperature. The increase in absorbance was measured relative to blank reactions. After construction of a concentration-response curve, the effect of disulfiram (0.1 mM in ethanol: 0.2% of final volume), an ALDH inhibitor, was examined in triplicate using the concentration of acetaldehyde (10 mM) that yielded maximal ALDH activity.

### Stress Protocol and Voluntary Alcohol Consumption

Mice were individually housed at 10 weeks of age and acclimatised to drinking from two freely available bottles of tap water for 1 week. Subsequently, one bottle was replaced with a 5% v/v ethanol solution for 3 weeks, which was increased to 10% v/v ethanol for the duration of the study. Alcohol/water bottles were randomly repositioned and weighed daily to determine preference and intake and mice were weighed weekly. Starting from the 23^rd^ day of access to 10% v/v ethanol, mice were restrained for 30 min/day between 1700 h and 1800 h for 7 consecutive days in modified 50 mL polypropylene tubes (Corning Inc., Corning, NY, USA) with holes drilled through the bottom and sides to permit breathing and airflow. One week after the last restraint stress, mice received 2 consecutive days of swim stress for 5 min/day between 1700 h and 1830 h in opaque buckets (19 cm diameter × 23 cm high) filled with 27°C tap water to a depth of 18 cm. Mice were maintained for a further 6 weeks until study conclusion.

### Statistics

SPSS software (Version 20; IBM Corp., Armonk, NY, USA) was used for all ANOVA statistical comparisons. GraphPad Prism (Version 5; GraphPad Software, Inc., La Jolla, CA, USA) was used for t-test statistical comparisons and to generate graphs. Baseline drinking for saccharin and alcohol studies is displayed as an average of the last 7 days of drinking (for alcohol, the last 7 days at each concentration). Data are expressed as means ± S.E.M.

## Results

### Rxfp3 KO and WT mice display similar taste perception and motivation to consume a palatable substance

**Fig 1:**
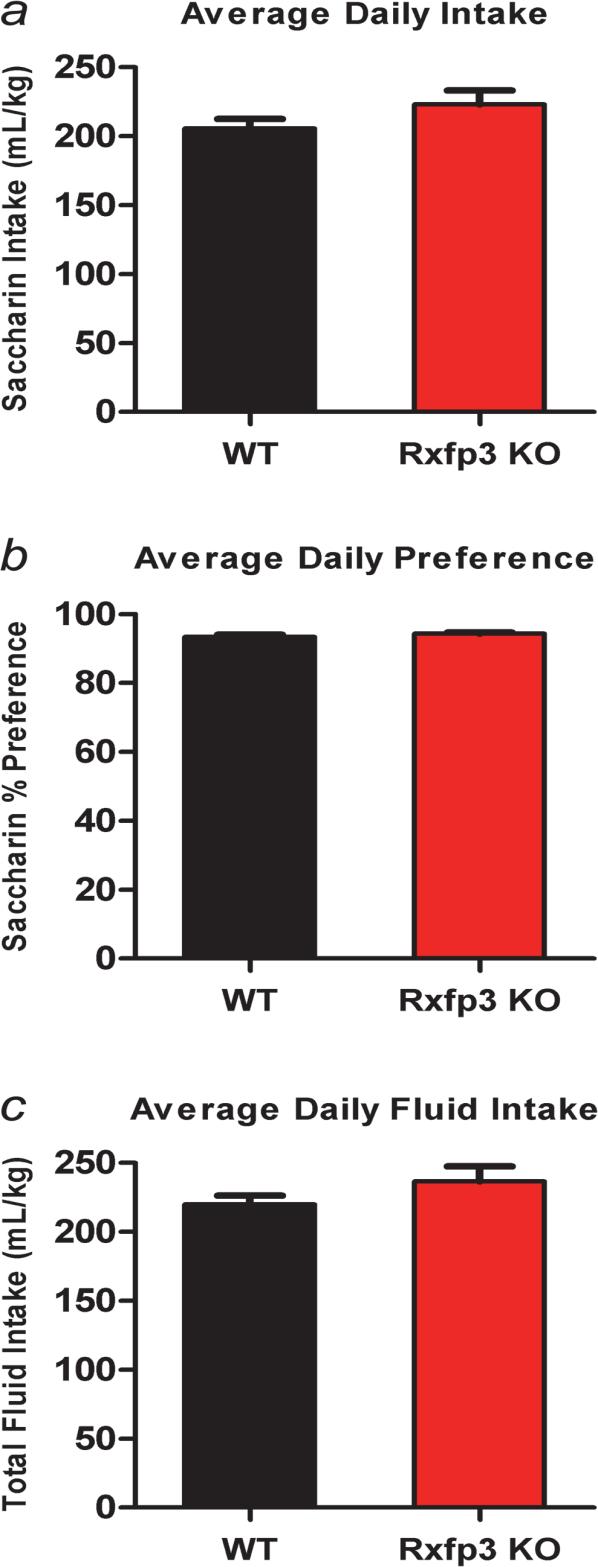
Saccharin Two-Bottle Free-Choice Drinking. Rxfp3 gene deletion does not alter taste perception or motivation to consume saccharin in mice. *(a)* Average daily saccharin consumption, *(b)* saccharin preference, and *(c)* total fluid intake of Rxfp3 KO mice (n = 11) and WT littermate controls (n = 14) over the final week of saccharin access. Data are presented as mean ± S.E.M.

A saccharin continuous access two-bottle choice test was used to evaluate taste perception and motivation to consume a palatable substance in WT (n = 14) and Rxfp3 KO (n = 11) mice. WT and Rxfp3 KO mice displayed similar average amounts of saccharin consumed per day (unpaired, two-tailed t-test, *t*_(23)_ = 1.50, *p* = 0.147; Fig 1*A*) and similar average daily preference for saccharin (unpaired, two-tailed t-test, *t*_(23)_ = 1.21, *p* = 0.238; Fig 1*B*). Furthermore, total fluid intake was comparable in both genotypes (unpaired, two-tailed t-test, *t*_(23)_ = 1.40, *p* = 0.175; Fig 1*C*).

### Rxfp3 KO and WT mice display similar baseline alcohol consumption and preference

**Fig 2:**
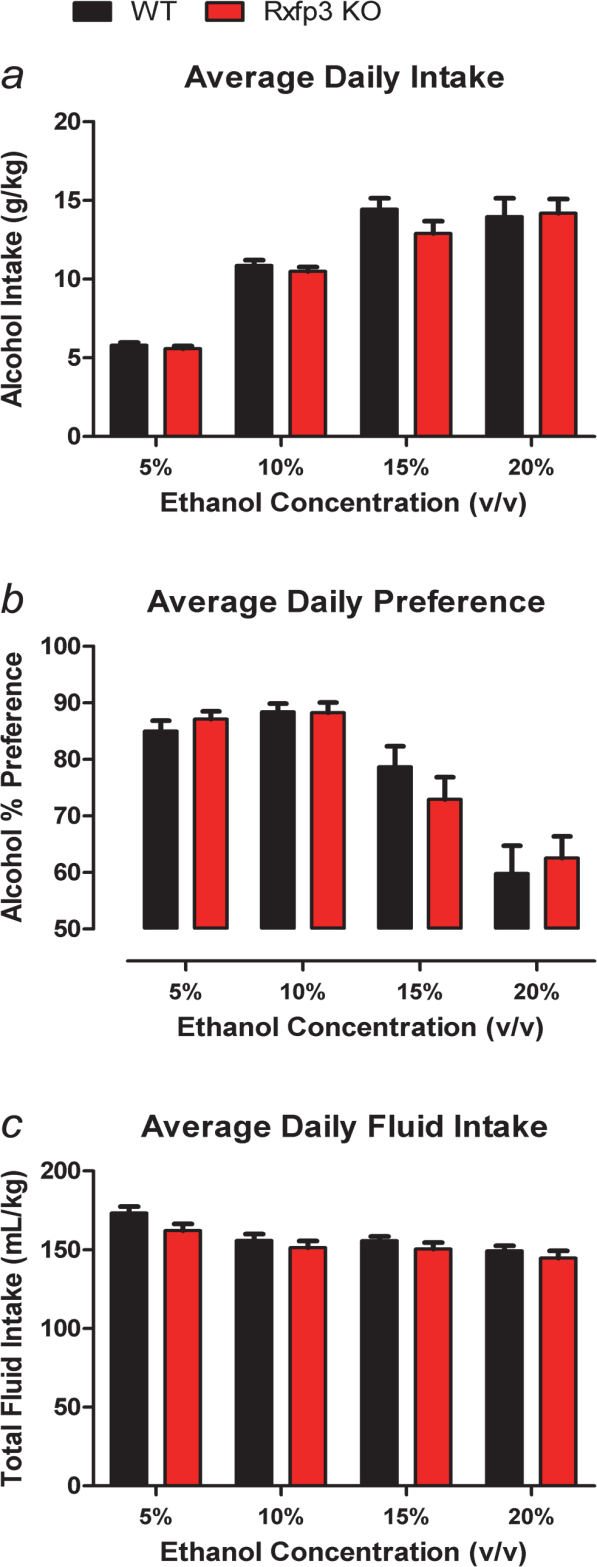
Alcohol Two-Bottle Free-Choice Drinking. Rxfp3 gene deletion does not impact baseline intake or preference for various ethanol solutions (5%-20% v/v) in the two-bottle choice paradigm. *(a)* Average daily alcohol consumption, *(b)* alcohol preference, and *(c)* total fluid intake of Rxfp3 KO mice (n = 16) and WT littermate controls (n = 17) over the final week of access to each ethanol solution. Data are presented as mean ± S.E.M.

Baseline alcohol consumption was measured in WT (n = 17) and Rxfp3 KO (n = 16) mice. In the continuous access two-bottle choice test, Rxfp3 KO mice displayed similar alcohol consumption and preference to WT littermate controls under ‘basal’ conditions, across increasing ethanol concentrations of 5, 10, 15 and 20% v/v. There were no genotype differences, overall or at different ethanol concentrations, in average daily amount of alcohol consumed (2-way RM ANOVA, main effect of genotype *F*_1,31_ = 0.512, *p* = 0.480; concentration × genotype interaction *F*_3,93_ = 0.988, *p* = 0.402; Fig 2*A*) or in average preference for alcohol (2-way RM ANOVA, main effect of genotype *F*_1,31_ = 0.006, *p* = 0.938; concentration × genotype interaction *F*_3,93_ = 1.20, *p* = 0.315; Fig 2*B*). However, increasing the concentration of available ethanol from 5 to 20% v/v increased average total amount of alcohol consumed per day from ~5 to 15 g/kg (main effect of concentration *F*_3,93_ = 104.2, *p*<0.0001; Fig 2*A*), and decreased average daily preference for alcohol over water from ~90 to 60% (main effect of concentration *F*_3,93_ = 48.93, *p*<0.0001; Fig 2*B*). Finally, there were no genotype differences overall or at each ethanol concentration in average daily total fluid intake (2-way RM ANOVA, main effect of genotype *F*_1,31_ = 1.553, *p* = 0.222; concentration × genotype interaction *F*_3,93_ = 2.170, *p* = 0.097; Fig 2*C*); although total fluid intake reduced slightly with increasing concentrations of available ethanol (main effect of concentration *F*_3,93_ = 63.75, *p*<0.0001; Fig 2*C*).

### Rxfp3 KO and WT mice display similar hepatic capacity to metabolise alcohol

**Fig 3:**
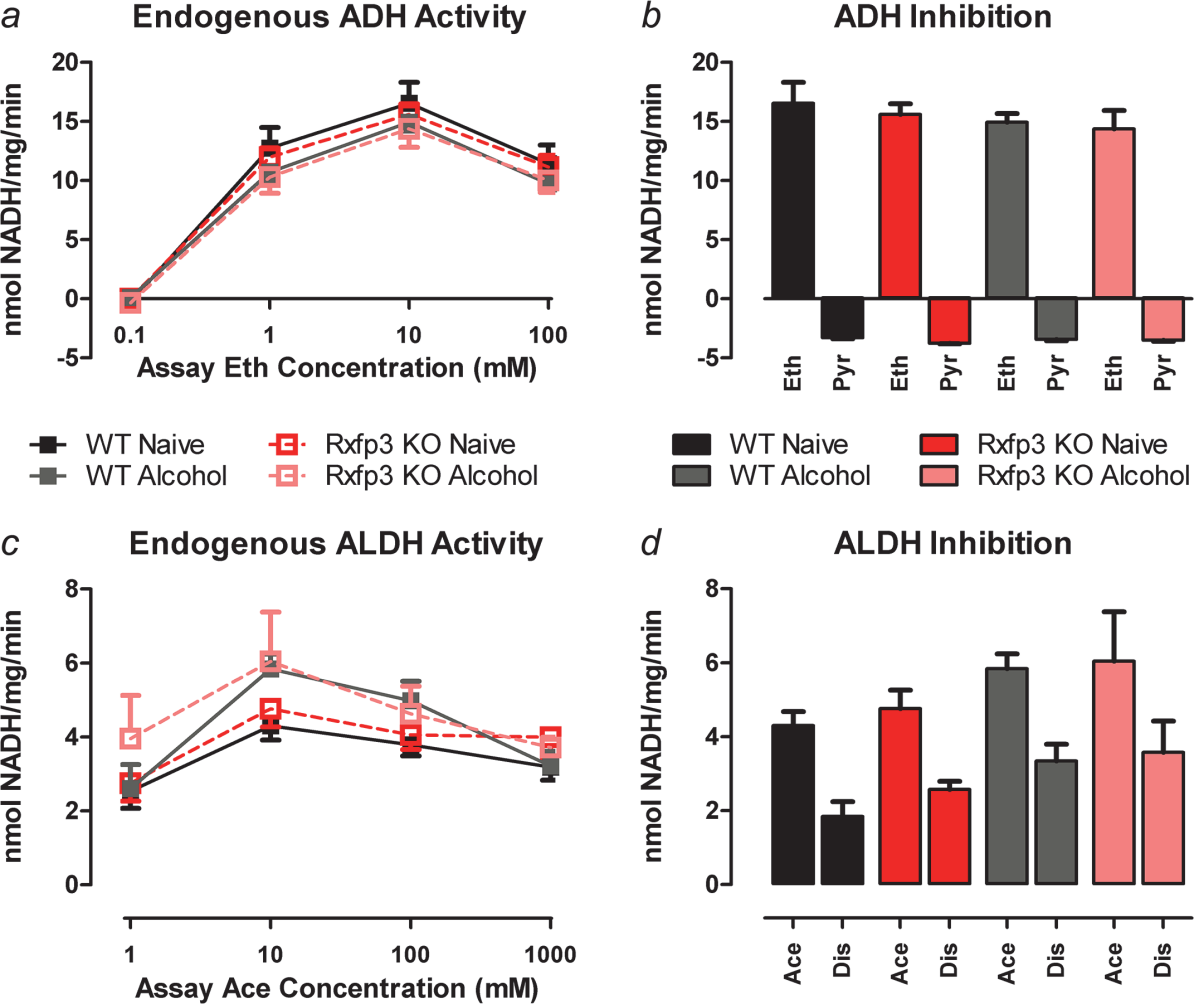
Alcohol and Aldehyde Dehydrogenase Activity. Endogenous ADH and ALDH activity in naive Rxfp3 KO (n = 7) and WT (n = 5) liver samples and liver preparations from Rxfp3 KO (n = 6) and WT (n = 6) mice that chronically consumed alcohol (5%-20% v/v) for 10 weeks, as measured by substrate-dependent increase in NADH absorbance. Nicotinamide adenine dinucleotide (β-NADH) was produced from the reduction of β-NAD+. *(a)* Increase in β-NADH as a result of ADH activity in the cytosolic fraction over various assay ethanol (Eth) concentrations. *(b)* Impact of pyrazole (Pyr; 10 mM), a specific ADH inhibitor, on the increase in β-NADH produced by the addition of 10 mM ethanol, the assay ethanol concentration that elicited maximal ADH activity. *(c)* Increase in β-NADH as a result of ALDH activity in the mitochondrial fraction over various assay acetaldehyde (Ace) concentrations. *(d)* Impact of disulfiram (Dis; 0.1 mM), a specific ALDH inhibitor, on the increase in β-NADH produced by the addition of 10 mM acetaldehyde, the assay acetaldehyde concentration that elicited maximal ALDH activity. Data are presented as mean ± S.E.M.

ADH activity was measured from liver samples taken from WT and Rxfp3 KO mice which were culled either naive (n = 5 WT/7 KO), or at the conclusion of the two-bottle choice experiment after chronic access to increasing concentrations (5%-20% v/v) of ethanol for 10 weeks (n = 6 WT/6 KO). No differences in endogenous ADH activity were observed between groups (3-way RM ANOVA, main effect of alcohol pre-treatment *F*_1,20_ = 1.363, *p* = 0.257; alcohol pre-treatment × genotype interaction *F*_1,20_ = 0.005, *p =* 0.944; alcohol pre-treatment × assay ethanol concentration interaction *F*_3,60_ = 3.453, *p* = 0.022; post-tests between alcohol pre-treatments, within assay ethanol concentrations, *p>*0.05; Fig 3*A*). Similarly, no differences were observed between genotypes (main effect of genotype *F*_1,20_ = 0.130, *p* = 0.722; genotype × assay ethanol concentration interaction *F*_3,60_ = 0.765, *p* = 0.518; Fig 3*A*). Endogenous ADH activity varied with assay ethanol concentration (main effect of assay ethanol concentration *F*_3,60_ = 947.614, *p*<0.0001; Fig 3*A*), with peak NADH absorbance occurring when 10 mM ethanol was used to initiate the reaction. Hence, the 10 mM ethanol concentration was used for a subsequent ADH inhibition assay, whereby pyrazole significantly reduced ethanol-induced ADH activity in both WT and Rxfp3 KO liver preparations (3-way RM ANOVA, main effect of pyrazole treatment *F*_1,20_ = 899.461, *p*<0.0001; Fig 3*B*).

No differences in ALDH activity were detected between groups or genotypes (3-way RM ANOVA, main effect of alcohol pre-treatment *F*_1,20_ = 1.540, *p* = 0.229; main effect of genotype *F*_1,20_ = 0.583, *p* = 0.454; alcohol pre-treatment × genotype interaction *F*_1,20_ = 0.0001, *p* = 0.992; alcohol pre-treatment × assay acetaldehyde concentration interaction *F*_3,60_ = 2.847, *p* = 0.045; genotype × assay acetaldehyde concentration interaction *F*_3,60_ = 0.944, *p* = 0.425; post-tests between alcohol pre-treatments and genotypes, within assay acetaldehyde concentrations, *p>*0.05; Fig 3*C*). As expected, endogenous ALDH activity varied with assay acetaldehyde concentration (main effect of assay acetaldehyde concentration *F*_3,60_ = 27.742, *p*<0.0001; Fig 3*C*), with peak NADH absorbance occurring when 10 mM acetaldehyde was used to initiate the reaction. 10 mM acetaldehyde was therefore used for subsequent studies to verify that ALDH activity was a major contributor to NADH absorbance. Disulfiram inhibited acetaldehyde-induced activity of ALDH by ~50% in both WT and Rxfp3 KO liver preparations (3-way RM ANOVA, main effect of disulfiram treatment *F*_1,20_ = 93.331, *p*<0.0001; Fig 3*D*).

### Rxfp3 KO mice display a stress-induced reduction in alcohol preference

**Fig 4:**
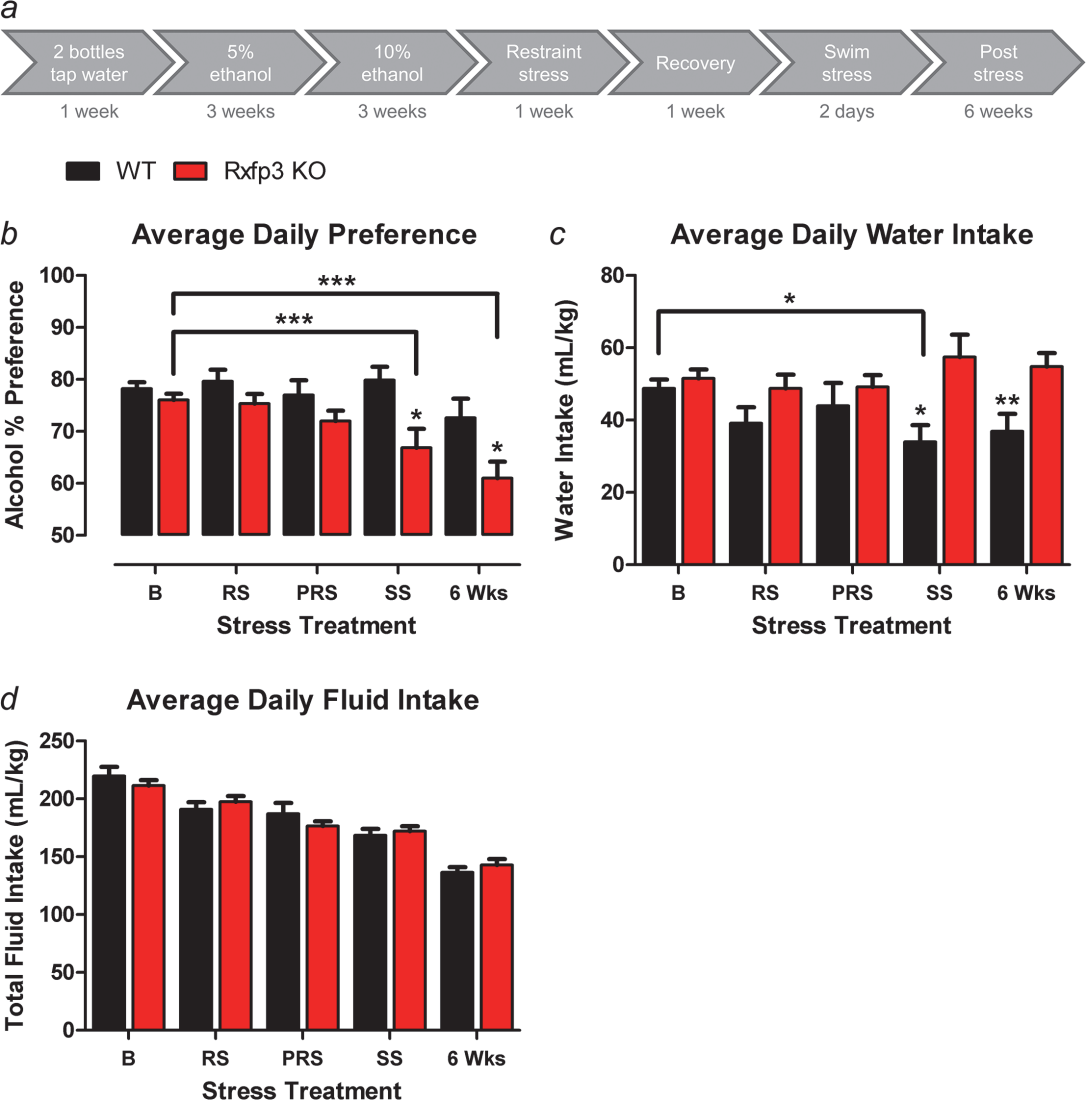
Stress Protocol and Stress-Related Alcohol Drinking. *(a)* Rxfp3 KO and WT mice were acclimatised to drinking from two bottles of tap water for 1 week. One bottle was subsequently replaced with a 5% v/v ethanol solution for 3 weeks, which was then increased to a 10% v/v ethanol solution for the duration of the experiment. Mice were permitted 3 weeks access to each ethanol solution prior to stress exposure to establish stable baseline drinking. From the 23^rd^ day of access to 10% v/v ethanol, mice were exposed to 7 consecutive days of restraint stress to determine the impact of multiple stress exposures on alcohol preference. After 1 week of recovery, mice were exposed to 2 consecutive days of swim stress as this stressor potently activates relaxin-3 systems and variable stress exposure models the human condition. Alcohol drinking was monitored for 6 weeks following exposure to the final stressor to assess persistent effects. *(b)* Rxfp3 gene deletion does not impact basal (B) alcohol preference but does result in reduced preference for a 10% v/v ethanol solution following restraint stress (RS), a post-restraint (PRS) period and swim stress (SS). Importantly, this phenotype persists for at least 6 weeks (Wks) following stress exposure. *(c)* WT (n = 9) mice significantly reduce water consumption during SS compared to B, resulting in less water intake compared to Rxfp3 KO (n = 20) mice, *(d)* whereas there is no difference in total fluid intake observed between Rxfp3 KO and WT littermate controls. Data are presented as mean ± S.E.M.

WT (n = 9) and Rxfp3 KO (n = 20) mice from Cohort 3, with access to 10% v/v ethanol, displayed similar alcohol preference and total fluid intake under basal (i.e. pre-stress) conditions, as observed in Cohort 2, indicating these findings are robust and reproducible (Fig 4*B* and 4*D*). Interestingly however, Rxfp3 KO mice exhibited a significant reduction in alcohol preference across stress treatments (2-way RM ANOVA, stress treatment × genotype interaction *F*_4,108_ = 3.463, *p* = 0.011; Bonferroni post-tests within Rxfp3 KO mice between stress treatments, baseline versus swim stress *p*<0.001, baseline versus 6 wks post-stress *p*<0.001; Fig 4*B*). This reduction in alcohol preference was not observed in WT mice (Bonferroni post-tests within WT mice between stress treatments, *p*>0.05; Fig 4*B*); and this discrepancy resulted in Rxfp3 KO mice displaying significantly less alcohol preference than WT littermates during swim stress, which persisted for at least 6 weeks after cessation of stress exposure (Bonferroni post-tests between genotypes during swim stress, *p* = 0.031; 6 wks *p* = 0.037; Fig 4*B*).

WT mice displayed a small but significant reduction in water intake across stress treatments, an effect that was not observed in Rxfp3 KO mice (2-way RM ANOVA, stress treatment × genotype interaction *F*_4,108_ = 3.746, *p* = 0.007; Bonferroni post-tests within WT mice between stress treatments, baseline versus swim stress *p*<0.05; Bonferroni post-tests within Rxfp3 KO mice between stress treatments *p*>0.05; Fig 4*C*). This discrepancy resulted in WT mice displaying significantly less water intake than Rxfp3 KO mice during swim stress, which persisted for 6 weeks (Bonferroni post-tests between genotypes during swim stress, *p* = 0.023; 6 wks *p* = 0.010; Fig 4*C*).

Analysis of total fluid intake (Fig 4*D*) revealed that the genotype differences in alcohol preference observed during swim stress and 6 weeks post-swim were not a result of differences in total fluid intake (2-way RM ANOVA, main effect of genotype *F*_1,27_ = 0.003, *p* = 0.954; stress treatment × genotype interaction *F*_4,108_ = 2.645, *p* = 0.037; post-tests between genotypes within stress treatments, *p*>0.05; Fig 4*D*). Reductions in total fluid intake were observed in WT and Rxfp3 KO mice across stress treatments (main effect of stress treatment *F*_4,108_ = 123.58, *p*<0.0001; Fig 4*D*).

## Discussion

These studies demonstrate that Rxfp3 KO mice exhibit similar baseline alcohol preference to WT littermates, but after repeated exposure to acute stressors (repeated restraint followed by swim stress) Rxfp3 KO mice reduced their alcohol preference, an effect not observed in WT mice. These observations suggest that RXFP3 signalling maintains high levels of alcohol preference during (and after) stress exposure, but does not appear to strongly regulate the primary reinforcing effects of alcohol. These data are in agreement with previous findings that icv and intra-BNST infusion of selective RXFP3 antagonists attenuate stress-induced reinstatement of alcohol-seeking in rat [18]. This phenotype persisted for at least 6 weeks after cessation of stress exposure, analogous to long-lasting alterations in alcohol drinking following stress in which CRF/CRF_1_ receptor signalling was implicated [37].

Mouse NI neurons express CRF_1_ [38], and it has been demonstrated in rat that relaxin-3 positive neurons in the NI express CRF_1_ [17, 25]. Additionally, relaxin-3 mRNA is up-regulated in rat NI following swim stress, which is attenuated by pre-treatment with a CRF_1_ receptor antagonist, suggesting relaxin-3 neurons are activated by swim stress in a CRF-dependent manner [16]. Importantly, conditional, brain-specific CRF_1_ receptor knockout (CRF_1_^NestinCre^) mice, which lack extra-hypothalamic CRF_1_ receptors, also displayed no difference in baseline alcohol consumption relative to WT littermates, but displayed attenuated social defeat stress-induced alcohol intake [10], highlighting the role of extra-hypothalamic CRF_1_ receptor signalling in stress-induced alcohol consumption, but not in the primary reinforcing properties of alcohol. Notably however, Molander et al. [10] reported an increase in alcohol consumption in CRF_1_^NestinCre^ mice and WT littermates following stress exposure, but to a lesser extent in CRF_1_^NestinCre^ mice, whereas our data reveal a stress-induced reduction in alcohol preference in Rxfp3 KO mice but unaltered alcohol preference in WT littermates.

The relationship between stress and alcohol intake is complex and the impact of stress on alcohol consumption in rodents is inconsistent in different studies. In fact, stress has been reported to increase, decrease or not change alcohol consumption in mice depending on the type of stressor used and the intensity, chronicity, predictability and controllability of the stressor [39]. Our findings, in agreement with other reports [40, 41], demonstrate that swim stress does not markedly alter alcohol two-bottle free-choice drinking in WT C57BL/6J mice, but suggest that intact relaxin-3/RXFP3 signalling may promote the maintenance of alcohol preference to relieve aversive states associated with acute and persistent anxiety produced by repeated stress exposure. Hence, alcohol preference was persistently attenuated in Rxfp3 KO mice after repeated stress exposure, in stark contrast to WT littermates. Long-lasting, stress-induced adaptations to alcohol drinking behaviour have been reported in mice [37] and relaxin-3/RXFP3 signalling is implicated in the regulation of chronic anxiety associated with prior stress exposure [33]. Importantly, mice in the present studies consumed ~10 g/kg/day of a 10% v/v ethanol solution, which is comparable to, even greater than amounts consumed by C57BL/6J mice in other reports [40, 41].

Central (icv) administration of an RXFP3 antagonist, R3(B1-22)R, significantly attenuated operant responding for alcohol in both alcohol-preferring (iP) and non-alcohol preferring Wistar rats, without impacting sucrose-responding in parallel experiments [18]. Additionally, icv administration of R3(B1-22)R significantly reduced both cue- and stress-induced reinstatement of alcohol-seeking, the effect being most marked for stress-induced relapse-like behaviour. Intra-BNST administration of R3(B1-22)R also significantly attenuated stress-induced reinstatement of alcohol-seeking, suggesting that RXFP3 signalling within the BNST is important for expression of stress-mediated alcohol-seeking behaviour in rat [18]. Importantly, the reduced alcohol preference in Rxfp3 KO mice following stress exposure observed in the present studies is in line with these pharmacological data in rat, and suggests that endogenous relaxin-3/RXFP3 signalling maintains stress-related responding for alcohol.

There were no significant differences between Rxfp3 KO and WT littermate mice in baseline alcohol preference or consumption across a range of ethanol concentrations (5%-20% v/v). Several factors may explain the discrepancy between baseline alcohol consumption in these mice and findings related to self-administration in rat. Firstly, although the relaxin-3/RXFP3 system in mouse is highly homologous to that in rat, there are species differences in distribution and expression levels of relaxin-3 and RXFP3 [23, 24]. Another key difference between these studies was the use of acute RXFP3 antagonist treatments in rat compared to germline Rxfp3 knockout in mice. Germline deletion of genes that encode proteins can induce developmental and other compensatory mechanisms that potentially mask behavioural consequences of the genetic manipulation [42–44]. It would be valuable, therefore, to repeat these studies using a conditional relaxin-3 or Rxfp3 KO model to circumvent any potential compensatory changes in RXFP3-related signalling. Also, there are clear differences between the operant conditioning paradigm used in earlier rat studies and the continuous access two-bottle choice paradigm employed in current studies. Self-administration allows only intermittent access to alcohol and the availability of an alcohol reward is contingent on an instrumental response. The two-bottle choice paradigm allows for continuous access to alcohol and alcohol availability is not response-dependent. Although alcohol preference and operant responding for alcohol likely share some underlying molecular mechanisms, alcohol drinking under two-bottle free-choice conditions may not fully translate to alcohol-reinforced drinking behaviour under operant conditions [45].

Importantly, the saccharin two-bottle free-choice study indicated no differences between Rxfp3 KO and WT mice in taste perception or baseline motivation to consume a palatable substance, as both genotypes displayed similar intake and preference for saccharin. Additionally, Rxfp3 KO and WT mice exhibited similar intake of tap water during acclimatisation to the paradigm and did not differ in total fluid intake under two-bottle choice conditions, suggesting similar fluid homeostasis. To our knowledge, RXFP3 is not expressed in mouse liver, but to assess hepatic liver enzyme activity, ADH and ALDH function was analysed in both naive mice and mice that consumed alcohol chronically for 10 weeks. Results indicated no differences in ADH or ALDH activity between any of the groups, suggesting that enzymatic pathways responsible for alcohol metabolism are intact in Rxfp3 KO mice. Consequently, the reduction in alcohol preference observed in Rxfp3 KO mice is likely due to a specific effect of RXFP3 signalling on stress responsiveness and the ability of stressors to regulate alcohol-seeking, rather than a secondary metabolic effect.

Alcohol has well established anxiolytic effects in humans and rodents, providing support for a ‘tension reduction’ hypothesis of alcohol use in certain circumstances [46–51]. The stress regimen employed in this study incorporated repeated exposure to acute restraint stress followed by two swim stress sessions. As canvassed above, acute, sub-chronic stress exposure during alcohol access has equivocal effects on alcohol consumption in rodents that depend on several characteristics of the stressor and animal strain [39]. Alcohol preference was significantly reduced in Rxfp3 KO mice towards the end of the stress exposure period and ongoing thereafter, so this effect might be due to a specific interaction of relaxin-3/RXFP3 signalling with swim stress or due to a cumulative stress load. Restraint and swim stress are both typically categorised as psychological stressors that, apart from obvious differences in locomotor activation, produce similar patterns of neuronal activation in the brain [52], recruit limbic nuclei, including the BNST [53, 54], and have a similar impact on HPA axis activation, as reflected by corticosterone release and immediate early gene activation [52, 55]. Consequently, it seems unlikely that swim stress would interact with relaxin-3/RXFP3 signalling more robustly than restraint stress to unmask the reduction in alcohol preference observed in Rxfp3 KO mice, particularly as both stressors engage the BNST, an area where RXFP3 signalling contributes to stress-induced reinstatement of alcohol-seeking [18]. The downward trend of preference during restraint stress that became significant during swim stress and thereafter, rather suggests cumulative stress load more likely contributes to the reduction in alcohol preference observed in Rxfp3 KO compared to WT mice. Exposure to variable, unpredictable stressors can alter future responses to stress as well as responsiveness to alcohol in rodents [56, 57]. Additionally, cumulative, variable stress exposure is relevant to alcohol dependence in the human condition as the number of stressful life events is predictive of first onset alcohol dependence [58], and variable stressors (e.g. family, work, health) are unavoidable in life.

We conclude that stress-reactive relaxin-3/RXFP3 signalling is likely involved in maintaining negatively reinforced alcohol preference to relieve persistent aversive states associated with exposure to variable stressors. Future studies are warranted to elucidate the circuit and molecular mechanisms that govern the interaction of relaxin-3/RXFP3 signalling with other systems to regulate complex behaviour.
